# PrP^Sc ^spreading patterns in the brain of sheep linked to different prion types

**DOI:** 10.1186/1297-9716-42-32

**Published:** 2011-02-15

**Authors:** Wiebke M Wemheuer, Sylvie L Benestad, Arne Wrede, Wilhelm E Wemheuer, Bertram Brenig, Bjørn Bratberg, Walter J Schulz-Schaeffer

**Affiliations:** 1 Prion and Dementia Research Unit, Department of Neuropathology, University Medical Center, Georg-August University, Robert-Koch Str. 40, 37075 Goettingen, Germany; 2 Norwegian Veterinary Institute, P.O. Box 750 Sentrum, 0106 Oslo, Norway; 3 Institute of Veterinary Medicine, Faculty for Agricultural Sciences, Georg-August University, Burckhardweg 2, 37075 Goettingen, Germany

## Abstract

Scrapie in sheep and goats has been known for more than 250 years and belongs nowadays to the so-called prion diseases that also include e.g. bovine spongiform encephalopathy in cattle (BSE) and Creutzfeldt-Jakob disease in humans. According to the prion hypothesis, the pathological isoform (PrP^Sc^) of the cellular prion protein (PrP^c^) comprises the essential, if not exclusive, component of the transmissible agent. Currently, two types of scrapie disease are known - classical and atypical/Nor98 scrapie. In the present study we examine 24 cases of classical and 25 cases of atypical/Nor98 scrapie with the sensitive PET blot method and validate the results with conventional immunohistochemistry. The sequential detection of PrP^Sc ^aggregates in the CNS of classical scrapie sheep implies that after neuroinvasion a spread from spinal cord and obex to the cerebellum, diencephalon and frontal cortex via the rostral brainstem takes place. We categorize the spread of PrP^Sc ^into four stages: the CNS entry stage, the brainstem stage, the cruciate sulcus stage and finally the basal ganglia stage. Such a sequential development of PrP^Sc ^was not detectable upon analysis of the present atypical/Nor98 scrapie cases. PrP^Sc ^distribution in one case of atypical/Nor98 scrapie in a presumably early disease phase suggests that the spread of PrP^Sc ^aggregates starts in the di- or telencephalon. In addition to the spontaneous generation of PrP^Sc^, an uptake of the infectious agent into the brain, that bypasses the brainstem and starts its accumulation in the thalamus, needs to be taken into consideration for atypical/Nor98 scrapie.

## Introduction

Scrapie in sheep and goats, which has been reported for more than 250 years [1], belongs to the transmissible spongiform encephalopathies (TSEs) - also known as prion diseases. This group of fatal diseases includes bovine spongiform encephalopathy (BSE) in cattle, chronic wasting disease (CWD) in deer and Creutzfeldt-Jakob disease (CJD) in humans. TSEs are characterized by the accumulation of protein aggregates, which are relatively stable against proteolysis. According to the prion hypothesis, a misfolded protein is the relevant part of the infectious agent [2]. It is widely accepted that this "proteinaceous infectious particle" is the pathological isoform of the physiological prion protein (PrP^c^) which is encoded by a cellular gene [3]. Recently, it has been shown that infectivity can be generated from a synthetic misfolded form of the prion protein [4]. Depending on the kind of prion disease, the pathological prion protein (PrP^Sc^) is detectable solely in the central nervous system (CNS) or may also be found in other tissues, especially in those of the lymphoreticular system (LRS) [5].

In the worldwide population of small ruminants, BSE and scrapie are considered to be the relevant TSEs affecting sheep and goats. Scrapie, however, is not a homogenous disease form, as demonstrated by the existence of several strains upon transmission to rodents [6] and the peculiar molecular properties of the sheep-passaged scrapie isolate CH1641 [7,8]. The discovery of a novel type of scrapie in Norway in 1998 (Nor98) that was clearly distinguishable from all previously reported forms of scrapie [9], and that was soon after detected in several other countries, added to the diversity of this TSE [10]. In our present work we concentrate on scrapie field cases that include cases of "classical" scrapie as well as "atypical"/Nor98 scrapie. Obvious differences exist between the two scrapie forms with regard to the epidemiology of the disease and the properties of the proteinaceous particle. The latter include Western blot profiles and the stability against denaturation and proteases [11-13]. The two forms of sheep scrapie also differ with regard to the genotypes affected. Amino acids at codon 136 (A/V), 154 (H/R) and 171 (H/Q/R) are considered to markedly influence susceptibility to classical scrapie; the most susceptible alleles are V_136_R_154_Q_171 _(VRQ) and A_136_R_154_Q_171 _(ARQ), while the A_136_R_154_R_171 _allele (ARR) seems to confer a certain resistance against the disease [14,15]. Atypical/Nor98 scrapie affects a number of genotypes, including the ARR allele, and animals with the AHQ allele or a Phenylalanin (F) instead of Leucin (L) at codon 141 in the ARQ allele are proportionally overrepresented [16-18].

The results of a number of case reports and studies have shown that the deposition form and distribution of PrP^Sc ^aggregates in atypical/Nor89 scrapie sheep are clearly distinct from classical scrapie; immunohistochemical methods and recently the sensitive PET blot method have been used for the detection of PrP^Sc ^in the ovine brain [9,19-23]. Formerly, the PET blot had only been used for the sensitive detection of PrP^Sc ^in extra-cerebral organs of classical scrapie sheep [24-27]. Surprisingly, the anatomical distribution of PrP^Sc ^in the ovine brain found in the literature is more thoroughly documented for atypical/Nor98 scrapie than for classical scrapie. Although the pathogenesis of classical scrapie is well-studied [28,29], detailed descriptions on how the infectious agent spreads once it has reached the brain seem to be lacking for both scrapie types. For classical scrapie, numerous reports exist on the different forms of PrP^Sc ^that can be found in the brain tissue and the presence of PrP^Sc ^aggregates in peripheral neural and non-neural tissues - at least in sheep carrying susceptible PrP genotypes. Also, the entry of the infectious agent into the CNS has been described thoroughly for field classical scrapie infections and has been shown to agree with the oral infection of sheep with BSE and scrapie as well as the oral infection of rodent models infected with scrapie [29-32]. The infectious agent apparently enters the CNS via the intermediolateral column of the thoracic spinal cord (Th_8 _- Th_10 _in natural scrapie infection) and the dorsal motor nucleus of the vagus nerve (DMNV) in the brainstem. Unfortunately, reports on the spread of ovine PrP^Sc ^from the brainstem into the brain are usually not very detailed. In atypical/Nor98 scrapie, most of the PrP^Sc ^load in affected sheep is found in the cerebellum and cerebrum. It still needs to be determined whether this novel disease is a sporadic prion disease or not. If sheep could acquire the disease from their environment, where would the infectious agent enter the CNS? The pattern of PrP^Sc ^deposition is apparently reproduced when atypical/Nor98 scrapie is transmitted from one sheep to another via intracerebral inoculation [33].

In this study the PrP^Sc ^deposition pattern in the CNS of 24 classical and 25 atypical/Nor98 field scrapie sheep was determined using the sensitive and specific PET blot method. Different amounts of PrP^Sc ^in the CNS of classical scrapie have been assigned to different stages of PrP^Sc ^spread into the brain, depending on the affected neuroanatomical structures.

## Materials and methods

### Material

The brains and, if available, the spinal cords as well as lymphatic tissue (tonsils and/or retropharyngeal lymph nodes) were collected from 49 scrapie field cases and 6 further sheep from scrapie-free flocks as controls. Scrapie positivity was diagnosed either ante mortem by tonsil biopsy or post mortem using the respective methods stipulated by the EU VO999/2001 at that time (samples were collected during a time span of 12 years). The scrapie-positive group included 19 German and 5 Norwegian sheep diagnosed with classical scrapie and 24 Norwegian atypical/Nor98 scrapie cases, plus one German atypical/Nor98 case. The control group was made up of six German sheep derived from scrapie-free flocks. The PrP genotypes were determined either by PCR and melting curve analysis [34] or by automated sequencing as described previously [9]. Further information on the individual animals including age, breed, genotype, presence of clinical signs and availability of LRS and spinal cord is listed in Table 1.

**Table 1:** Genotype, age, breed, the presence of clinical signs and the availability of lymphatic tissue and spinal cord of the individual sheep

	Genotype	Breed	Age in months	Lymphatic tissue available	Spinal cord available	Clinical signs present
**Classical scrapie cases**						

	ARQ/ARQ	G.M./B.M. crossbreed	~42	yes	yes	no
	
	ARQ/ARQ	G.M./B.M. crossbreed	~24	yes	yes	no
	
	ARQ/ARQ	G.M./B.M. crossbreed	>48	yes	yes	no
	
	ARQ/ARQ	Black headed Mutton	~72	yes	yes	no
	
	ARQ/ARQ	German Merino	~60	yes	yes	yes
	
	ARQ/ARQ	German Merino	>48	yes	yes	yes
	
	ARQ/ARQ	G.M./B.M. crossbreed	>48	yes	yes	yes
	
	ARQ/ARQ	G.M./B.M. crossbreed	unknown	yes	no	unknown
	
	ARQ/ARQ	G.M./B.M. crossbreed	~48	yes	no	unknown
	
	ARQ/ARQ	Black headed Mutton	unknown	yes	no	unknown
	
	ARQ/ARQ	B.M./Mountain sheep crossbreed	27	yes	yes	yes
	
	ARQ/ARQ	G.M./B.M./Mountain sheep crossbreed	25	yes	yes	yes
	
	ARQ/ARQ	B.M./Mountain sheep crossbreed	unknown	yes	yes	yes
	
	ARQ/ARQ	B.M./Mountain sheep crossbreed	29	yes	yes	no
	
	ARQ/ARQ	B.M./Mountain sheep crossbreed	38	yes	yes	yes
	
	VRQ/ARQ	Texel	unknown	yes	no	unknown
	
	VRQ/ARQ	Norwegian pelt sheep	~24	yes	no	unknown
	
	VRQ/ARQ	Steigar sheep	~42	no	no	unknown
	
	VRQ/ARQ	Texel	unknown	yes	no	unknown
	
	VRQ/ARQ	Steigar sheep	unknown	no	no	unknown
	
	VRQ/ARQ	Texel/Mountain sheep crossbreed	32	yes	yes	yes
	
	VRQ/ARH	Texel	30	yes	yes	no
	
	VRQ/ARH	Steigar sheep	unknown	yes	no	unknown
	
	VRQ/ARH	Texel	unknown	yes	no	unknown

**Atypical/Nor98 scrapie cases**						

	AFRQ/ARQ	Spæl sheep	~78	no	no	uncertain
	
	AFRQ/AHQ	Suffolk/Rygja/Steigar crossbreed	~72	no	yes	yes
	
	ARQ/AHQ	German Merino	unknown	yes	no	unknown
	
	AHQ/AHQ	Steigar sheep	~48	yes	yes	yes
	
	AHQ/AHQ	Spæl sheep	~72	yes	no	yes
	
	AHQ/AHQ	Norwegian white sheep	~84	yes	no	yes
	
	AHQ/AHQ	Spæl sheep	~42	yes	yes	uncertain
	
	AHQ/AHQ	Spæl sheep	~48	yes	no	yes
	
	AHQ/AHQ	Spæl sheep	~72	no	no	yes
	
	AHQ/ARH	Norwegian white sheep	~120	yes	no	yes
	
	AHQ/AFRQ	Dala sheep	~84	no	yes	yes
	
	AHQ/AFRQ	Steigar sheep	~60	no	no	yes
	
	AFRQ/AFRQ	Norwegian white sheep	~60	yes	no	uncertain
	
	AFRQ/AFRQ	Dala sheep	~36	yes	yes	yes
	
	AFRQ/AFRQ	Norwegian white sheep	~78	yes	no	yes
	
	AFRQ/AFRQ	Rygja/Dala crossbreed	~84	yes	no	uncertain
	
	AFRQ/AFRQ	Dala sheep	~108	yes	no	no
	
	AHQ/ARR	Spæl sheep	~72	no	no	yes
	
	AHQ/ARR	Norwegian white sheep	~96	yes	no	no
	
	AHQ/ARR	Dala sheep	~72	yes	no	uncertain
	
	AHQ/ARR	Dala sheep	~84	no	yes	no
	
	ARR/AFRQ	Norwegian white sheep	~96	no	no	unknown
	
	ARR/AFRQ	Norwegian white sheep	~66	yes	no	no
	
	ARR/ARR	Steigar	~84	no	yes	no
	
	unknown	Rygja/Dala crossbreed	unknown	no	no	no

**Control cases**						

	ARQ/ARQ	Skudde	unknown	yes	no	no
	
	ARR/ARH	Leine	30	yes	no	no
	
	ARR/ARR	German Merino	~132	yes	no	no
	
	ARR/ARR	Leine	~60	yes	no	no
	
	ARR/ARR	Leine	~96	yes	no	no
	
	ARR/ARR	Leine	>48	yes	no	no

B.M. = blackheaded mutton, G.M. = German Merino

Depending on the circumstances under which the samples were collected, the post mortem times of tissues varied between 2 h and 4 days. Usually one half of the brain**/**tonsil/lymph node was fixed in 4% buffered formaldehyde, cut into slices and embedded in paraffin within five to seven days, while the other half was frozen and stored at -80 °C.

### Histopathology

One to three μm-thick CNS**/**lymphatic tissue sections were cut, collected on silane-coated glass slides and stained with haematoxylin and eosin (H&E). Brain sections were also stained with Luxol Fast Blue then counterstained by periodic acid Schiff` reagent (LFB/PAS) for the orientation and discrimination of neuronal nuclei and neural tracts.

### PET blot

The PET blot procedure followed the protocol as described previously [23,35] using the monoclonal antibody (mAb) P4 (R-Biopharm, Darmstadt, Germany), which had proved to give the best results regarding sensitivity and specificity for the detection of PrP^Sc ^in classical and atypical/Nor98 sheep scrapie [23]. In brief, immunolabeling of PrP^Sc ^was performed after a 1-3 μm tissue section had been placed on a nitrocellulose membrane (0.45 μm, Bio-Rad, Hercules, CA, USA) which was then deparaffinized and rehydrated. This was followed by treatment with proteinase K (250 μg/mL; Sigma-Aldrich, MO, USA) overnight at 56 °C and the decontamination of the membranes in 4 M guanidine thiocyanate (GdnSCN) for 30 min. Membranes were blocked with 0.2% casein in PBS containing 1% Tween before the primary antibody (mAbP4) was applied 1:5000 in TBST. An alkaline phosphatase-coupled goat-anti-mouse antibody (Dako, Glostrup, Denmark) and the formazan-reaction with NBT/BCIP were used to visualize the result. Thorough rinsing of the membranes with TBST was required between the different steps.

### Immunohistochemistry

Tissue sections on silane-coated glass slides were stained with one of the primary mAbs P4, L42 (R-Biopharm, Darmstadt, Germany), F89/160.1.5 (Veterinary Medical Research and Developement, Pullman, WA, USA), and 12F10 (kindly provided by W Bodemer and D Motzkus, German Primate Center), which were used 1:500 in combination with an alkaline phosphatase-coupled goat anti-mouse antibody (Dako) and neufuchsine as chromogene as described previously [23]. Alternatively, a commercially available kit from Dako (Envision AEC, Glostrup, Denmark) was applied by using mAb F89/160.1.5 at a dilution of 1:2000 in combination with the mAb 2G11 (1:200, kindly provided by J Grosclaude (INRA, Jouy-en-Josas, France)).

### Examination and evaluation of immunolabelled sections

From each sheep all available sections of the CNS and the LRS were examined with the PET blot, and the intensity of the PrP^Sc ^staining as well as the forms and distribution of the PrP^Sc ^deposition were evaluated. The presence of PrP^Sc ^deposits and the deposition forms in CNS and LRS sections were verified by immunohistochemistry. This was usually done using either mAb P4 (German cases) or mAb F89/160.1.5 in combination with mAb 2G11 (Norwegian cases), but if considered necessary immunohistochemistry was repeated with further antibodies as stated above. The intensity of PrP^Sc ^deposits in the PET blots was evaluated on a scale of 0.5 to 4 (0 = no PrP^Sc ^deposits visible; 0.5 = very little indefinable deposits; 1 = very little distinct PrP^Sc ^deposits; 1.5 = little distinct PrP^Sc ^deposits; 2 = moderate PrP^Sc ^deposits, all deposition forms well distinguishable; 2.5 = moderate to pronounced PrP^Sc ^deposits, all deposition forms well distinguishable; 3 = pronounced PrP^Sc ^deposits, deposition forms partly interfere with each other; 3.5 = pronounced PrP^Sc ^deposits, deposition forms interfere with each other; 4: maximal PrP^Sc ^deposits, deposition forms interfere with each other). The value system of the scale itself was established and agreed on by two independent persons that routinely evaluate PET blots.

### Western blot analysis

Ten percent tissue homogenates (wt/vol) were either prepared in PBS containing 0.5% desoxycholic acid sodium salt (DOC) using glass grinding tubes and pestles or 20% homogenates were obtained by the standard sampling procedure of the TeSeE Western Blot Kit (Bio-Rad, Hercules, CA, USA).

Twenty percent homogenates were processed using the TeSeE sheep/goat Western Blot Kit according to the manufacturer's instructions. The antibody P4 was added at a dilution of 1:1000 to the primary antibody of the kit. Ten percent homogenates were subjected to a different protocol using homemade 15% acrylamid gels, a 0.45 μm nitrocellulose (NC) membrane (Bio-Rad) for semi-dry blotting and mAbP4 (1:2000). The membrane was treated with 4 M GdnSCN and blocked with 0.2% casein in PBS including 1% Tween for 30 min respectively before the primary antibody was applied overnight at 4 °C. An HRP-conjugated goat anti-mouse antibody (Dako, Carpintera, CA, USA) and Super Signal Femto West Maximum Sensitivity Substrate (Perbio, Erembodegem, Belgium) were used to visualize the result on x-ray film. The molecular size of PrP^Sc ^was compared only within one system.

## Results

### Western blot

In all sheep that had been classified as atypical/Nor98 scrapie cases, the characteristic small fragment of 11-12 kDa [9] was present in CNS tissue samples after proteinase K digestion. Hereof the typical triplet pattern of 18-30 kDa in all classical scrapie sheep was clearly distinguishable. We usually used CNS tissue for Western blotting to determine the molecular profile. Only in one sheep with classical scrapie PrP^Sc ^were amounts in the brainstem so minimal that lymphatic tissue was needed to perform a valid Western blot. To ensure that Western blot PrP^Sc ^patterns of different tissues were comparable in one sheep, lymphatic tissues of further sheep with classical scrapie (from this study) were examined as well.

### PET blot and immunohistochemistry

Disease-associated prion protein could be identified in the CNS of all scrapie sheep with the PET blot and there was no PrP^Sc ^detectable in the tissues of the negative control group. As previously described, immunohistochemical methods were able to confirm the presence of PrP^Sc ^deposits in all sheep except for one atypical/Nor98 case, despite using of a panel of antibodies [23].

Immunolabeling with the PET blot method allowed the identification of a number of deposition forms of PrP^Sc ^in the CNS and all were confirmed by immunohistochemical methods.

**Figure 1:**
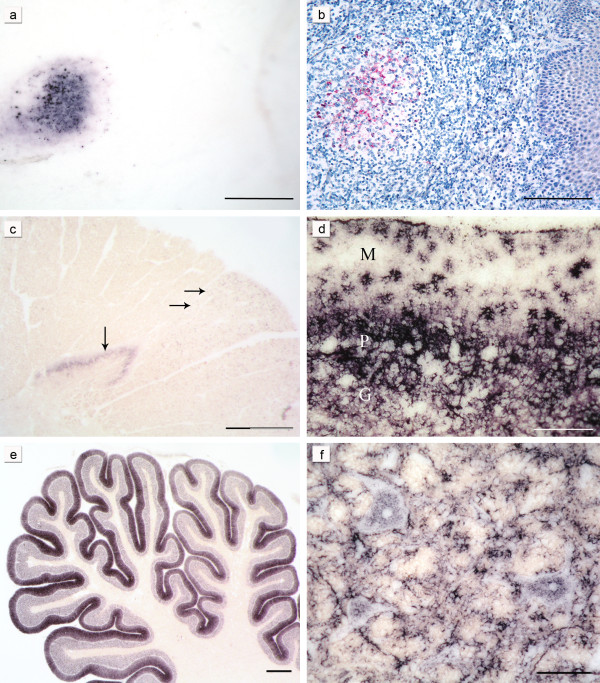
**Characteristic PrP^Sc^ deposition patterns in classical and atypical/Nor98 scrapie:** The same lymph follicle in the tonsil of a sheep with classical scrapie is shown stained either with the PET blot method (a) or conventional immunohistochemistry (b) (both mAb P4, bars = 250 μm). In the cervical spinal cord segment of a sheep with atypical/Nor98 scrapie (c) stained with the PET blot method (mAb P4, bar = 800 μm) synaptic PrP^Sc ^aggregates are present in the substantia gelatinosa (vertical arrow) and granular PrP^Sc ^in the corticospinal tract (horizontal arrows). In the cerebellar cortex of a classical scrapie sheep stained with the PET blot (d) complex PrP^Sc ^deposits are visible. Glia-associated PrP^Sc ^deposits take a stellate form in the molecular layer (mAb P4, bar = 150 μm; M = molecular layer, P = layer of Purkinje cells, G = granular cell layer). In the majority of the atypical/Nor98 scrapie sheep the cerebellar cortices show a more intense staining of the molecular layer than the granular layer (e) (PET blot, mAb P4; bar 600 μm). Intraneuronal, perineuronal and glia-associated PrP^Sc ^aggregates (f) in the reticular formation of a classical scrapie sheep (PET blot, mAb P4; bar = 50 μm). Tissue sections derived from sheep with the genotypes VRQ/ARH (a,b), AHQ/AHQ (c,e) and ARQ/ARQ (d,f).

As described before [23] PrP^Sc ^was detectable in the LRS tissue of all classical scrapie sheep where it was present, but in none of the atypical/Nor98 scrapie animals with available LRS tissue could PrP^Sc ^be found (for availability of lymphatic tissue see Table 1). Figure 1a/b shows PET blot and immunohistochemical staining of the PrP^Sc ^aggregates in the follicle of a tonsil derived from a classical scrapie case.

#### Deposition forms

Intra- and perineuronal PrP^Sc ^aggregates were found with the PET blot solely in classical scrapie (Figure 1f), as were subpial, subenpendymal, and perivascular deposits. Extra neuronal PrP^Sc ^aggregates in the brains of sheep affected by classical scrapie often had a ramified appearance and were found in grey and white matter structures (Figure 1d and 1f). They were addressed as glia-associated PrP^Sc ^aggregates and found to be relatively conspicuous in the cerebellar molecular layer where they took a stellate form [36] (Figure 1d).

In contrast, PrP^Sc ^aggregates found in the white matter of atypical/Nor98 scrapie sheep were always well-defined granules that varied a bit in size and were occasionally arranged like pearls on a string. The latter deposition form could also be observed in classical cases, but here also linear PrP^Sc ^was sometimes present. PrP^Sc ^deposits in the grey matter of atypical/Nor98 scrapie cases generally showed a fine granular pattern, also termed "synaptic/reticular" in human TSEs rather than "fine granular" [12,37] (see Figure 1c). In some atypical/Nor98 scrapie cases, larger plaque-like aggregates could be seen in the substantia nigra, basal ganglia, thalamic nuclei and white matter. However, a differentiation between real plaques (amyloid) and plaque-like deposits is not possible with immunohistochemical detection methods or with the PET blot method as demonstrated before [38].

A discrimination between globular and punctuate deposits in the white matter of atypical/Nor98 cases [11,21] was irreproducible with the PET blot, which is why the term "granular" for the PrP^Sc ^deposits that were present in the white matter was chosen. Punctuate PrP^Sc ^deposits, comprising smaller aggregates than granular PrP^Sc ^deposits but more defined than the reticular PrP^Sc ^aggregates, were detected in the grey matter of classical scrapie sheep.

Small deviations in the composition of the complex deposition pattern could not be related to genotypes in the sheep examined.

#### Distribution of PrP^Sc ^in the CNS

##### Sequential appearance of PrP^Sc ^distribution in the CNS of classical scrapie sheep

**Figure 2:**
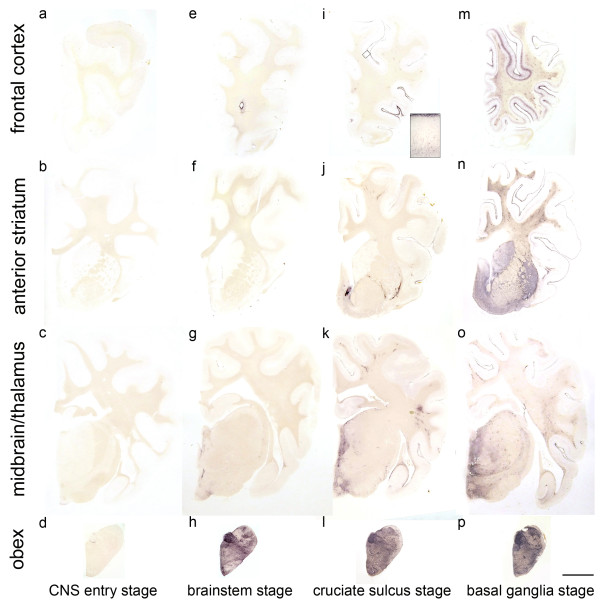
**Classification of the PrPSc spread during disease development in classical scrapie.**The examined classical scrapie cases classified into four stages of PrP spread  according to certain affected neuroanatomical sites (PET blots, mAb P4). In the **CNS entry stage (a - d)** only discrete PrP^Sc ^deposits are visible in the obex region, while in the **brainstem stage (e - h)** PrP^Sc ^aggregates are clearly visible in the brainstem and start to appear in more rostral structures. Once PrP^Sc ^deposits can be found in the deep cortical layers of the frontal cortex (i), the **cruciate sulcus stage (i - l)** is reached. In the **basal ganglia stage**, intense deposits in basal ganglia and thalamic nuclei can be found **(m - p)**. Brain sections shown for the first, third and fourth stage derived from sheep with the genotype ARQ/ARQ while the sheep whose brain sections are depicted in the brainstem stage carried the genotype ARH/VRQ (bar = 5 mm).

**Figure 3:**
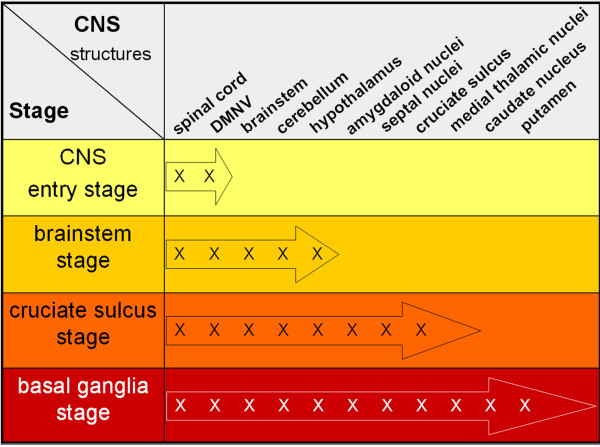
** Progression of classical scrapie in the brain shown for certain affected neuroanatomical sites**. The colour code agrees with the one in Figure 5.

**Figure 4:**
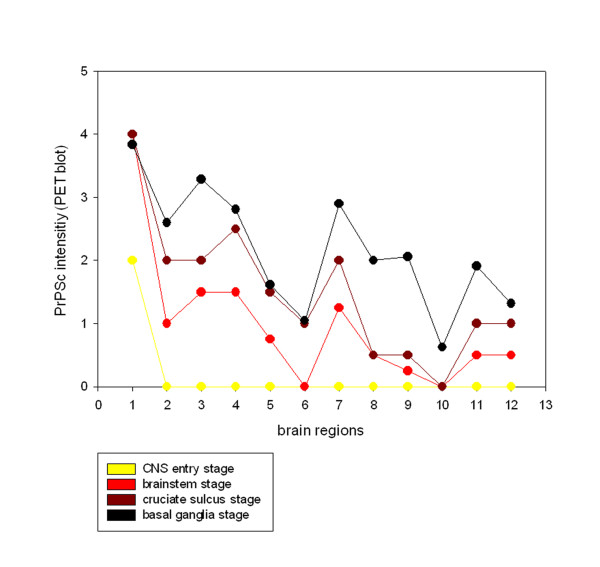
**Accumulation of PrP^Sc^ in different brain regions during disease progression:** The four stages of the examined classical scrapie cases are depicted in four overlying graphs that illustrate how PrP^Sc ^aggregates (PET blot method, mAb P4) are increasingly accumulated in the brains from caudal (left) to rostral (right). Evaluation of PrP^Sc ^intensity was performed on a scale from 0.5 - 4 (see material and methods) and shown for the following brain areas: 1 dorsal motor nucleus of the vagus nerve (DMNV), 2 inferior olive, 3 dorsal tegmental nucleus, 4 cerebellar molecular layer, 5 cerebellar granular layer, 6 cerebral peduncle, 7 central grey (mesencephalon), 8 caudate nucleus, 9 ventral pallidum, 10 rostral commissure, 11 cruciate sulcus, 12 frontal white matter.

**Figure 5:**
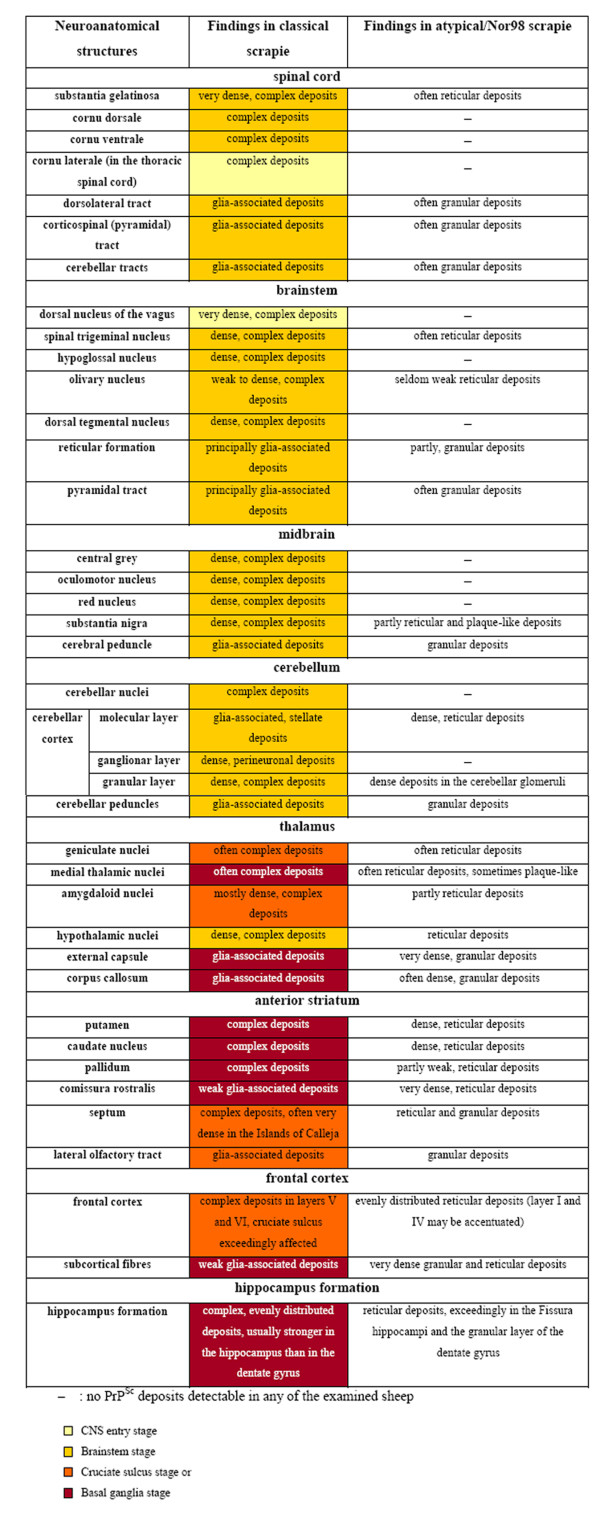
**Form and appearance of PrP^Sc ^deposits in classical and atypical/Nor98 scrapie sheep presented for representative CNS regions**. As a sequential development of PrP^Sc ^distribution could not be observed upon analysis of the present atypical/Nor98 scrapie cases, no coding colours were used for the results of this scrapie type in contrast to classical scrapie sheep. PrP^Sc ^deposits are detectable in the respective location in classical scrapie sheep belonging to the stages of spread indicated in the bottom of the figure using the same colour code as in Figure 3.

To determine the sequential appearance of PrP^Sc ^in the CNS, all field cases of classical scrapie were subjected to a thorough examination regarding the anatomical structures affected by PrP^Sc ^deposition. In the following, all cases were arranged according to the amount of PrP^Sc ^they had accumulated in total, and the occurrence of PrP^Sc ^in a panel of 127 neuroanatomical loci was compared between the cases. From this evaluation arose a classification of the classical scrapie cases into four stages of PrP^Sc ^spread in the CNS (see Figures 2, 3, 4 and 5). Criteria for these turned out to be certain neuroanatomical structures whose involvement marked a stage, meaning that the respective structure accumulated PrP^Sc ^aggregates (with a minimal score of 1) in all animals belonging to this stage and the following stage/stages. They are described in detail below and visualized in Figures 3 and 4.

**CNS entry stage:** One sheep showed only few discrete PrP^Sc ^deposits in the brain that were restricted to the dorsal motor nucleus of the vagus nerve (DMNV), the solitary tract nucleus and the spinal trigeminal tract in the brainstem. Further PrP^Sc ^aggregates could be detected in the substantia intermedialis lateralis and centralis of the thoracic spinal cord. This first stage, where PrP^Sc ^is detectable only in these CNS areas, can be considered the "CNS entry stage" in accordance with studies of other authors who have monitored the ascension of PrP^Sc ^from the intestines to the CNS [29,30].

**Brainstem stage: **In the second stage, all segments of the spinal cord and all nuclei of the obex region accumulate PrP^Sc ^which also disseminates to the more rostral parts of the medulla; this may therefore be called "brainstem stage". In the caudal medulla the cellulae marginales and substantia gelatinosa of the spinal trigeminal tract nucleus show a very intense staining. The mesencephalon and thalamus display discrete PrP^Sc ^deposits which are generally found to be subpial and/or perivascular while the mamillary body, habenular nuclei and the hypothalamic nuclei accumulate substantial amounts of PrP^Sc^. The cerebellar nuclei accumulate PrP^Sc ^if the rostral medulla is largely involved and focal deposits of PrP^Sc ^are visible in the cerebellar cortex.

**Cruciate sulcus stage:** During the next stage, the mesencephalon, amygdaloid nuclei, septal nuclei, optic tract, cerebral peduncle, hippocampus formation, frontal cortex and subcortical white matter are increasingly affected. Regarding the frontal cortex, it is notably the sulcus cruciatus - and in a number of cases only this part of the cortex - that accumulates PrP^Sc ^in its deeper cortical layers (see Figure 2i). This stage is therefore designated "cruciate sulcus stage". PrP^Sc ^deposits in the cerebellar cortex are not yet evenly distributed.

**Basal ganglia stage: **In the final stage, PrP^Sc ^deposits can be seen also in the medial thalamic nuclei (mediodorsal, ventrolateral, ventral posterior and anterior group), the corpora geniculata and the basal ganglia. A positive staining for PrP^Sc ^in the latter determines a classical case in our definition for the "basal ganglia stage". The white matter also displays remarkable amounts of PrP^Sc^, which are strongly linked to perivascular distribution.

All stages are depicted in Figures 2, 3 and 4 and the stage at which PrP^Sc ^reaches a respective neuroanatomical site is indicated in Figure 5 using a colour code. In the sheep examined in this study we could not find any influence of the different genotypes on the neuroanatomical distribution of PrP^Sc ^aggregates.

##### Comparison of PrP^Sc ^deposition patterns in classical and atypical/Nor98 scrapie

PrP^Sc ^deposits in atypical/Nor98 scrapie cases were examined and evaluated in the same way as with the classical field cases.

**Figure 6:**
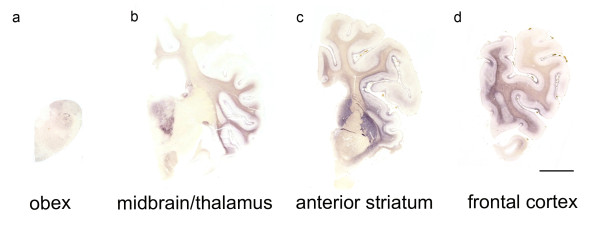
**PrP^Sc^ distribution in the brain of atypical/Nor98 scrapie:** Brain sections of an atypical/Nor98 scrapie case stained with the PET blot (mAb P4) have a different PrP^Sc ^distribution than the ones of classical scrapie cases as shown in Figure 2 (bar = 5 mm). Brain section derived from a sheep with the genotype ARQ/AHQ.

**Figure 7:**
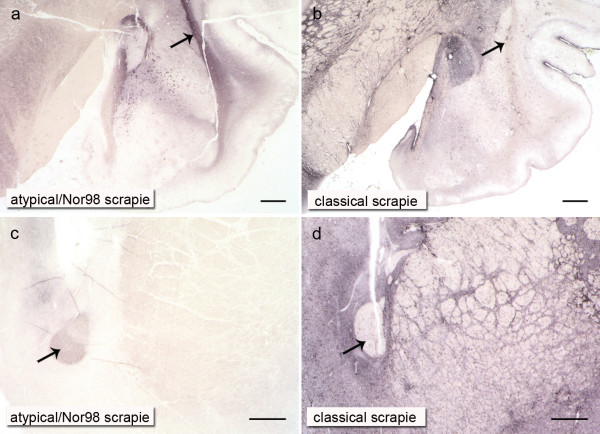
**Differences in the neuroanatomical distribution of PrP^Sc ^deposit in atypical/Nor98 and classical sheep scrapie: **In atypical/Nor98 scrapie, white matter structures like the external capsule or rostral commissure contain substantially more PrP^Sc ^than the subcortical nuclei or basal ganglia respectively (a and c), whereas this is the reverse in classical scrapie (b and d). The external capsule (a and b) and the rostral commissure (c and d) are marked with arrows (mAb P4, bars = 1 mm). Tissue derived from sheep with the genotypes ARQ/AHQ (a), ARQ/ARQ (b and d) and AHQ/AFRQ (c).

In contrast to the classical scrapie cases, differentiating distribution/spread stages of PrP^Sc ^in the CNS was not feasible with the atypical/Nor98 scrapie cases. In Figure 6, the same brain sections that illustrate the different stages of classical scrapie in Figure 2 are depicted for a case of atypical/Nor98 scrapie. In all atypical/Nor98 scrapie sheep, where brainstem material was available (*n *= 15) apart from one (see below), PrP^Sc ^aggregates were detectable in the rhombencephalon and mesencephalon. Regularly affected neuroanatomical structures were the spinal trigeminal nucleus, reticular formation, pyramid, pontine fibres, substantia nigra and cerebral peduncle. In the spinal cord the corticospinal tract and substantia gelatinosa accumulated PrP^Sc ^in most cases (Figure 1c). Certain grey matter structures such as the DMNV, hypoglossal nucleus, dorsal tegmental nucleus, oculomotor nucleus, red nucleus and central grey of the mesencephalon, never displayed any PrP^Sc ^in the examined atypical/Nor98 scrapie cases. These listed neuroanatomical sites, however, accumulated large amounts of PrP^Sc ^in the respective stage of PrP^Sc ^distribution in the CNS of classical scrapie sheep as explained above (Figures 2, 3, 4 and 5). There were no PrP^Sc ^aggregates detectable in the cerebellar nuclei of the examined atypical/Nor98 scrapie cases, in contrast to the classical scrapie cases as described above. The synaptic or reticular PrP^Sc ^staining pattern in the cerebellar cortex of atypical/Nor98 scrapie sheep was in most cases more intense in the molecular than in the granular layer (Figure 1e). Intra- and extracellular complex PrP^Sc ^aggregates in the cerebellar cortex of classical scrapie sheep were predominantly present in the granular layer and surrounding the Purkinje cells; the molecular layer displayed mainly glia-associated PrP^Sc ^deposits that took a stellate form (Figure 1d). The cerebellar peduncles and white matter of the cerebellum itself showed PrP^Sc ^aggregates for both scrapie types. In the diencephalon of most atypical/Nor98 scrapie sheep, the corpora geniculata, medial thalamic nuclei and reticular nucleus accumulated PrP^Sc ^aggregates. In all atypical/Nor98 cases where the anterior striatum could be examined (*n *= 14), PrP^Sc ^deposits were also present in the caudate nucleus and putamen. The white matter of diencephalon and telencephalon showed PrP^Sc ^deposits in both types of sheep scrapie. In atypical/Nor98 scrapie, these were mainly confined to the subcortical fibres and certain white matter tracts, e.g. the corpus callosum or the commissura rostralis (Figure 7d, arrow), while the distribution in classical scrapie was more disseminated.

There was one case in which PrP^Sc ^deposits were detectable with the PET blot only in the supratentorial (cerebral) brain structures and to a very small degree in the cerebellar cortex. The brainstem, including midbrain and spinal cord, were completely spared in this case, which was eventually considered to represent an early stage of atypical/Nor98 scrapie [23].

In Figure 7 the contrasts in PrP^Sc ^intensity existing in the grey and white matter between the two types of scrapie are demonstrated in a case of atypical/Nor98 scrapie and a classical scrapie case of the "basal ganglia stage": in classical scrapie it is the centromedial amygdaloid nuclei (Figure 7b) as well as the septal nuclei and basal ganglia (Figure 7d) that show substantially more PrP^Sc ^than the external capsule (Figure 7b, arrow) and the rostral commissure (Figure 7d, arrow). In atypical/Nor98 scrapie, this principle turns out to be exactly the opposite, with the external capsule (Figure 7a, arrow) and the rostral commissure (Figure 7c, arrow) accumulating rather intense PrP^Sc ^deposits in contrast to the adjacent grey matter.

The lateral olfactory tract displayed PrP^Sc ^aggregates in both scrapie types with the respective PrP^Sc ^deposition patterns described above. Yet, the Islands of Calleja - clusters of neuronal granular cells in the olfactory tubercle - showed dense PrP^Sc ^deposits solely in classical scrapie cases and were completely devoid of PrP^Sc ^in atypical/Nor98 scrapie sheep. Regarding the hippocampus formation in classical scrapie cases, there was usually a more intense staining of the hippocampus and the fissura hippocampi compared to the dentate gyrus. In contrast to the atypical/Nor98 scrapie cases, there was no obvious accentuation of any layers. Atypical/Nor98 scrapie sheep showed a rather intense PrP^Sc ^staining of the granular layer of the dentate gyrus, the fissura hippocampi and the interconnective fibres between hippocampus and alveus (similar to the subcortical white matter) in comparison to the adjacent layers. The pyramid layer of the hippocampus appeared to be completely devoid of PrP^Sc ^deposits. The intensity of PrP^Sc ^staining in a single case was usually in agreement with the intensity of PrP^Sc ^deposits that could be found in the cerebral cortex of both scrapie types. As mentioned above, the complex PrP^Sc ^aggregates in classical scrapie were mainly confined to the deeper cortical layers (laminae V and VI) while reticular/synaptic PrP^Sc ^deposits in the cortices of atypical/Nor98 scrapie sheep were distributed more evenly, although an accentuation of laminae I and IV could be noted in some cases. Like in classical scrapie, differences regarding the distribution of PrP^Sc ^deposition could not be related to genotypes.

## Discussion

In this study 24 cases of classical and 25 cases of atypical/Nor98 scrapie cases were examined with the PET blot method, focusing on the similarities and differences in the distribution of PrP^Sc ^deposits that were detectable with this method. Recently the PET blot has been shown to provide a sensitive and specific detection of PrP^Sc ^in both types of sheep scrapie in the same manner as had been previously shown for human, bovine and rodent neuronal and non-neuronal tissues [30,35,38-41]. The high sensitivity of this method allows PrP^Sc ^deposits to be detected even in FFI patients where conventional immunohistochemistry fails to detect them, and contrasts with Western blotting, which requires up to 1 g of tissue equivalent [42]. The PET blot provides, apart from its sensitivity and specificity, a good overview of where to find PrP^Sc ^in a brain section (Figure 2), as no counterstaining is necessary. The fine resolution of the immunolabeling gives a good impression of the structures that accumulate PrP^Sc ^(Figure 1f), but the general delineation of the single cell is better with immunohistochemistry, which is why these two methods complement each other in a sensible way.

### Neuroinvasion and spread of PrP^Sc ^in the ovine brain

In this study, we also give a more detailed account of how the disease-associated PrP aggregates seem to spread in the CNS tissue of sheep infected with classical scrapie. The sequential detection of PrP^Sc ^aggregates in the CNS of classical scrapie sheep implies that a cell-to-cell spread takes place from the entry sites in the spinal cord and obex to the cerebellum, diencephalon and frontal cortex via the rostral brainstem. From these entry sites we conclude that the vagus nerve for the DMNV and sympathetic fibres for the spinal cord are the structures that transport the infectious agent to the CNS. This is very similar to the results obtained in hamsters after oral inoculation with the 263K scrapie strain [43]. The cerebellum may also receive PrP^Sc ^via the cerebellar tracts of the spinal cord. Noticeable perivascular PrP^Sc ^deposition in the brains of scrapie-affected sheep also raises the possibility that the infectious agent reaches the brain via the haematogenous route [44]. The distribution of brain metastases in humans reflects a haematogenous entry into the brain as it is proportional to the cerebral blood flow per area. From this one can conclude that a general PrP^Sc ^uptake from the blood would cause quite a different cerebral distribution pattern of PrP^Sc ^deposits than we observed [45]. There are three other possible explanations for the perivascular accumulation of PrP^Sc ^aggregates in classical scrapie. Cells of glial origin, e.g. microglia, might use the blood vessels as a structural lead for their movement and carry PrP^Sc ^molecules with them, possibly also distributing them among the astrocytes forming the blood brain barrier (BBB). This would be a vascular spread in the broader sense. As a second possibility, microglia cells that have incorporated PrP^Sc ^move to the blood vessels in order to dispose of the aggregates and this leads to a perivascular deposition of the aggregates. Another way for PrP^Sc ^to reach blood vessels could be that they spread via sympathetic nerve fibres of the Plexus nervorum perivascularis. Haematogenous neuroinvasion has also been discussed with regard to the circumventricular organs (CVOs) due to the fact that these are usually affected in scrapie-infected sheep and that they are not protected by the BBB [46]. The possibility that the CVOs might be in contact with PrP^Sc ^from the blood during the pathogenesis of the disease cannot be excluded, but our results argue against a major involvement of the CVOs in neuroinvasion. In a very early case the DMNV was affected, but the area postrema and further CVOs were devoid of PrP^Sc ^(Figure 2d). This agrees again with the results obtained for the oral infection of hamsters with scrapie [30].

In contrast to the classical scrapie cases, a sequential development of PrP^Sc ^distribution cannot be seen upon analysis of the present atypical/Nor98 scrapie cases. PrP^Sc ^distribution in one sheep of presumably early disease phase suggests that the aggregation of PrP^Sc ^has its origin in the di- or telencephalon. A spontaneous genesis of misfolded PrP could arise in the cerebral cortex. On the other hand, an ascending spread of the infectious agent that bypasses the brainstem and enters the CNS via sensible nerve fibres should be taken into consideration, e.g. proprioceptive fibres [47] or the spinothalamic tract. This would lead to further spreading of PrP^Sc ^from the thalamic nuclei to the cerebellar and cerebral cortex and from these to the brainstem and spinal cord, e.g. via the corticospinal tract.

### Where does the spread of Nor98- PrP^Sc ^start in the brain?

It has been speculated by Nentwig et al. [48] that the PrP^Sc ^deposits and histopathologic lesions in atypical/Nor98 scrapie possibly evolve from the cerebrum to the cerebellum and the brainstem, but according to their examination of six sheep brains, this concept would not explain the PrP^Sc ^distribution in one sheep where they found PrP^Sc ^mainly in the cerebellum. However, immunohistochemistry - as used by these authors - is sometimes not able to detect the fine reticular deposits, e.g. seen in the cortex of Creutzfeldt-Jakob disease type 1, especially in the rare VV1 subtype [49]. The sensitive PET blot method, in contrast, is able to visualize these reticular deposits [38]. PrP^Sc ^deposits in one case described by Nentwig et al. could have therefore simply been missed in the cortex by immunohistochemistry. If this proved to be correct, according to the argumentation of Nentwig et al., PrP^Sc ^deposition and histopathologic lesions could indeed evolve from the cerebrum into the cerebellum and the brainstem. The 15 whole brains of atypical/Nor98 scrapie sheep examined by Moore et al. [21] should accordingly represent more or less the final stage of disease, as PrP^Sc ^can be generally found in all parts of the brain, including the brainstem. In other reports on the occurrence of atypical/Nor98 scrapie, cases have been described in which no PrP^Sc ^was detectable by immunohistochemistry in the obex region at all, but in the cerebellum and cerebrum [50-54]. If the misfolding of PrP^c ^in atypical/Nor98 scrapie does really start in the cerebrum it is obvious why early stages are not present in the worldwide pool of preserved atypical/Nor98 brains, as only the sampling of brainstem and cerebellum is compulsory in small ruminants according to EU regulations. Thus the question of whether PrP^Sc ^accumulation might start sporadically in the cerebrum - and if so, at one or more sites at the same time? - cannot be resolved by this or any other current study using field cases of atypical/Nor98 scrapie. This situation is comparable to the one with CJD type 1, where a spontaneous misfolding of PrP^c ^in the cerebral cortex and a caudal spread from there is assumed, but not proven [55]. The incidence of atypical**/**Nor98 in sheep is higher than that of CJD in humans [17]. A case control study of atypical/Nor98 scrapie has shown that animal movement does not seem to be a factor for the transmission of atypical/Nor98 scrapie between flocks; thus if sheep were to acquire this prion disease from their environment, its contagiousness would indeed be very low [56]. It has been speculated that this might be due to the relatively low protease stability, which could also explain the lack of intracellular PrP^Sc ^deposits [33].

There are certainly small differences between the PrP^Sc ^distribution detected by Moore et al. [21] in their described atypical/Nor98 scrapie cases and the ones revealed here by the PET blot method. For instance, in the present atypical/Nor98 scrapie material, PrP^Sc ^was never detectable in the cerebellar nuclei. Also, the affected parts of the hippocampus appear to be different. This might be due to differences in the treatment of tissue, the methods and/or differences in the antibodies used (mAb2G11 versus mAbP4). As previously reported, perineuronal staining has also been detected for the substantia nigra in some atypical**/**Nor98 scrapie sheep using immunohistochemistry [11], whereas in our study only plaque-like PrP^Sc ^deposits could be seen in this neuroanatomical structure. Similarly, neuronal deposits could be found in many affected sites of classical scrapie, but in contrast to previous publications [22], neither PET blot nor immunohistochemistry revealed PrP^Sc ^in the Purkinje cells of the cerebellum. It is known that especially intraneuronal immunoreactivity needs to be interpreted with caution [10]. However, the congruence between previous reports on PrP^Sc ^deposition patterns and the present results is obvious.

## Conclusion

In summary, this study gives a basic description of PrP^Sc ^deposition patterns in classical as compared to atypical/Nor98 scrapie cases using the sensitive and specific PET blot method. We were able show a sequential appearance of PrP^Sc ^aggregates in the CNS of sheep with classical scrapie, but not in atypical/Nor98 scrapie. The four emerging stages of spread in classical scrapie were defined by the accumulation of PrP^Sc ^in certain neuroanatomical structures. These structures accumulated PrP^Sc ^aggregates in all animals belonging to this stage and the following stage/stages. Further conclusions drawn from this study regarding atypical/Nor98 scrapie might help in future to elucidate its origin and potentially related prion disease types like Creutzfeldt-Jakob disease type 1.

## Competing interests

The authors declare that they have no competing interests.

## Authors' contributions

WMW carried out the PET blot studies, participated in immunohistochemistry, Western blot, tissue acquisition and the design of the study and drafted the manuscript. SLB participated in immunohistochemistry, Western blot, tissue acquisition and design of the study and co-edited the manuscript. AW, WEW, BeB and BjB participated in tissue acquisition and diagnosing the cases. WJSS conceived the study, participated in its design and coordination and co-edited the manuscript. All authors read and approved the final manuscript.
